# Japan’s cancer survivorship guidelines for exercise and physical activity

**DOI:** 10.1093/jjco/hyae126

**Published:** 2024-09-19

**Authors:** Katsunori Tsuji, Hiroyuki Sasai, Kosuke Kiyohara, Yoshio Nakata, Hiroki Nishiwaki, Takahisa Ohta, Eisuke Ochi, Toshimi Takano, Noriatsu Tatematsu, Yutaka J Matsuoka

**Affiliations:** Sports Research Center, Hosei University, Kawasaki-shi, Kanagawa, Japan; Research Team for Promoting Independence and Mental Health, Tokyo Metropolitan Institute for Geriatrics and Gerontology, Itabashi-ku, Tokyo, Japan; Department of Food Science, Otsuma Women’s University, Chiyoda-ku, Tokyo, Japan; Institute of Health and Sport Sciences, University of Tsukuba, Tsukuba-shi, Ibaraki, Japan; Division of Nephrology, Department of Internal Medicine, Showa University Fujigaoka Hospital, Yokohama-shi, Kanagawa, Japan; Showa University Research Administration Center (SURAC), Showa University, Shinagawa-ku, Tokyo, Japan; Institute of Clinical Epidemiology (iCE), Showa University, Shinagawa-ku, Tokyo, Japan; Research Team for Promoting Independence and Mental Health, Tokyo Metropolitan Institute for Geriatrics and Gerontology, Itabashi-ku, Tokyo, Japan; Faculty of Bioscience and Applied Chemistry, Hosei University, Koganei-shi, Tokyo, Japan; Graduate School of Sports and Health Studies, Hosei University, Machida-shi, Tokyo, Japan; Breast Medical Oncology, The Cancer Institute Hospital of Japanese Foundation for Cancer Research, Koto-ku, Tokyo, Japan; Department of Integrated Health Sciences, Nagoya University Graduate School of Medicine, Nagoya-shi, Aichi, Japan; Division of Quality Assurance Programs, National Cancer Center Institute for Cancer Control, Chuo-ku, Tokyo, Japan; Division of Cancer Information Service, National Cancer Center Institute for Cancer Control, Chuo-ku, Tokyo, Japan; Division of Policy Evaluation, National Cancer Center Institute for Cancer Control, Chuo-ku, Tokyo, Japan

**Keywords:** cancer survivorship guideline, physical activity, exercise, cancer survivor, Delphi process

## Abstract

**Objective:**

This research aimed to establish the inaugural evidence-based cancer survivorship guidelines for Japan, with a particular focus on exercise and physical activity, in order to enhance health outcomes for cancer survivors.

**Methods:**

A panel of experts, including oncologists, physicians, exercise scientists, epidemiologists and patient advocates, utilized a modified Delphi process and systematic reviews to establish consensus on exercise recommendations for cancer survivors. The panel focused on setting the objectives of the Clinical Practice Guidelines and addressing crucial clinical issues in Japan. Recommendations were formulated based on the strength and certainty of evidence, the benefit–harm balance and patient values and preferences.

**Results:**

The panel formulated exercise recommendations for cancer survivors of two age groups: 18–64 years and ≥65 years. The recommendations for both age groups are consistent, emphasizing the importance of regular exercise and physical activity tailored to individual capabilities and health conditions. The guidelines underline the benefits of exercise in improving the overall health and quality of life of cancer survivors. This consensus on exercise recommendations marks a significant step in the development of comprehensive cancer survivorship guidelines in Japan, with potential implications for improving clinical outcomes and advancing research in cancer survivorship.

**Conclusions:**

These guidelines will serve as a critical resource for cancer survivors, highlighting exercise as a key component of survivorship care, and for clinicians, in recommending appropriate physical activities to improve survivor health and well-being.

## Teaser

This study establishes the first evidence-based exercise and physical activity guidelines for cancer survivorship in Japan, proposing recommendations to enhance health and quality of life in cancer survivors.

## Introduction

Physical activity is widely recognized as a critical component of cancer survivorship care, with several guidelines emphasizing its importance for cancer survivors. The American Society of Clinical Oncology (ASCO) and the American Cancer Society (ACS) jointly issued guidelines in 2003 (1), which were revised in 2012 (2), that recommend healthy weight management, a physically active lifestyle and a healthy diet for cancer survivors. Similarly, the American College of Sports Medicine (ACSM) released the ‘Exercise Guidelines for Cancer Survivors’ in 2010, advocating for at least 150 minutes of exercise per week, including resistance training and stretching exercises (3). A Cochrane Review published in 2012 reinforces these recommendations, highlighting the substantial benefits of exercise in improving the quality of life (QoL) of adult cancer survivors (4). Furthermore, recent systematic reviews have confirmed that exercise is both safe and feasible during cancer treatment and can contribute positively to multiple health outcomes, including the alleviation of depressive symptoms (5). In 2018, a multidisciplinary roundtable comprising representatives from ACS, ACSM, the Centers for Disease Control, the National Cancer Institute and the National Comprehensive Cancer Network, among others, convened to update the ‘Exercise Guidelines for Cancer Survivors’, which were published in 2019 (6). These guidelines state that ‘exercise...[was] generally safe for cancer survivors and that every survivor should avoid inactivity. Enough evidence was available to conclude that specific doses of aerobic, combined aerobic plus resistance training, and/or resistance training could improve common cancer-related health outcomes, including anxiety, depressive symptoms, fatigue, physical functioning, and health-related quality of life’. Despite these well-established benefits, global adherence to physical activity guidelines among cancer survivors remains suboptimal. For example, the U.S.-based Health Information National Trends Survey (HINTS) conducted in 2003 reported that 45% of a sample of 619 cancer survivors and 32% of surveyed breast cancer survivors had not engaged in regular physical activity since developing cancer (7). Similarly, a nationwide ACS lifestyle survey of 9105 cancer survivors found that only 37% of breast cancer survivors followed the physical activity recommendations (8).

Even with the availability of comprehensive global guidelines, Japan still faces considerable challenges in ensuring that cancer survivors engage in the recommended levels of physical activity. In Japan, cancer represents a major public health issue, with one out of every two people developing cancer in their lifetime. According to 2020 data, there were 534 814 cases of cancer in men and 410 238 cases in women, totaling 945 055 cases (9). Advances in cancer therapy have improved the 5-year relative survival rate in Japan from 54.3% in the late 1990s to 64.1% in recent years (10). Consequently, an increasing number of cancer survivors are living with or beyond cancer while remaining socially active. However, despite the well-documented benefits of physical activity and exercise, many cancer survivors in Japan do not adhere to the recommended levels of physical activity. Furthermore, existing guidelines from abroad do not adequately address the diverse needs of cancer survivors. Specifically, they lack tailored recommendations based on age, habitual exercise levels and other individual factors, which may limit their effectiveness in promoting physical activity among different subgroups of survivors. This gap highlights the need for more personalized guidelines that cater to the unique characteristics of Japanese cancer survivors.

To address these gaps, our study aimed to develop Japan’s inaugural evidence-based cancer survivorship guidelines focusing on exercise and physical activity. These guidelines target cancer survivors aged 18 years and older who are disease-free or have stable disease. We developed targeted exercise recommendations by distinguishing between habitual and non-habitual exercisers and between different age groups (18–64 years and 65 years or older). By clarifying these recommendations, we aim to provide more personalized and effective guidance for physical activity in cancer survivorship, potentially improving clinical outcomes and advancing research in this field. These guidelines can supplement existing disease-specific guidelines prepared by relevant academic associations, thereby providing a comprehensive framework for improving the overall health and QoL of cancer survivors in Japan.

## Guideline questions

In developing these guidelines, we proposed the following two clinical questions (CQs): (CQ1) Should exercise be recommended to cancer survivors aged 18–64 years who are not habitual exercisers? (CQ2) Should exercise be recommended to cancer survivors aged ≥65 years who are not habitual exercisers?

## Methods

### Guideline development process

#### Establishment and objectives

The ‘National Cancer Center Cancer Survivorship Guidelines’ Development Oversight Committee (GDOC), comprising 22 members, was established in October 2018 (11). The GDOC held meetings and conducted online surveys aimed at generating consensus on the overall scope of comprehensive cancer survivorship guidelines as well as the matters to be addressed in the Clinical Practice Guidelines (hereafter referred to as ‘the CPG’ or ‘the Guidelines’) proposed here. Specifically, the target population of the CPG was defined as ‘patients aged 18 years or older who have been diagnosed with cancer and who are currently disease-free or who have stable disease’. Meanwhile, ‘individuals undergoing cancer pharmacotherapy (excluding maintenance chemotherapy for blood cancers and hormone therapy to prevent recurrence of breast, prostate and other cancers) or undergoing treatment for recurrent, metastatic or terminal cancers’ were excluded. The intended users of the CPG were defined as ‘all healthcare professionals; primarily those providing care to cancer survivors diagnosed at 18 years or older who are currently disease-free or who have stable disease’, and the intended clinical settings were defined as ‘settings used by all healthcare professionals; primarily those providing care to cancer survivors diagnosed at 18 years or older who are currently disease-free or who have stable disease’. Furthermore, ‘maintaining a high level of physical activity’ was identified as an important clinical issue to be prioritized in the CPG.

#### Drafting recommendations and Delphi methods

The ‘National Cancer Center Cancer Survivorship Guidelines for Exercise and Physical Activity’ Clinical Practice Guideline Development Group (CPG-DG) was subsequently formed, and the CPG-DG Administrative Office was established in April 2019 in accordance with the ‘Medical Information Network Distribution Service (MINDS) Manual for Developing Clinical Practice Guidelines 2017’. Comprising experts in the fields of exercise, rehabilitation, oncology, general medicine and mental health, the CPG-DG held a total of 15 in-person or online meetings and engaged in email-based discussions between April 2019 and September 2020. The final panel meeting held in September 2020 also featured a discussion that included the GDOC patient advocates in order to incorporate the views of non-experts. Recommendations were selected upon reaching an 80% consensus among GDOC members through online voting on draft recommendations prepared by the CPG-DG based on the modified Delphi method (12). If a consensus could not be reached, the GDOC held meetings to repeat the review process. Recommendations were adopted after considering factors such as patient values, patient burden, and risk of bias in the studies subject to systematic review, focusing on the level of evidence and the benefit–harm balance of significant outcomes of the CQ. The guideline recommendations were then opened to review and comment by experts and laypeople by soliciting external evaluation and public comment. These comments were considered when finalizing the recommendations. The expert panel members were responsible for reviewing and approving the final version of the guidelines. The approved guidelines were then submitted to the GDOC.

In developing the CPG, we referenced the ‘MINDS Manual for Developing Clinical Practice Guidelines 2017’. However, due to practical constraints, some adjustments were necessary. For example, the literature search focused on key databases in order to manage resources effectively. In addition, although patients were not involved at the beginning, we incorporated their input during the recommendation phase and included two patient representatives in the external review committee to ensure diverse perspectives. Given the rapid advances in scientific knowledge, new evidence can emerge in the time it takes for information to be created and published (or read). This information may not be up-to-date and may not reflect the latest evidence. Moreover, information that pertains exclusively to a specific topic is typically not applicable to other interventions, diseases or disease stages.

#### Systematic literature review

##### Literature search strategy

The CPG recommendations were developed based on thorough reviews of studies identified in literature searches of the databases PubMed, Cochrane Central, Ichushi-Web (an online database operated by the Japan Medical Abstracts Society) and SPORTDiscus. The searches employed designated search queries and handsearching to identify relevant studies in a comprehensive manner. Detailed search strategies, including the search terms, are provided in Supplementary Table 1. Only data obtained from randomized controlled trials (RCTs) were considered. Our systematic review protocol was registered with PROSPERO, an international prospective register of systematic reviews (registration number: CRD42020206402). Individual RCTs were selected based on the following inclusion criteria.

(i) Target population: cancer survivors (individuals aged ≥18 years who have been diagnosed with cancer and who are currently disease-free or who have stable disease). (The definition of ‘cancer survivor’ shall adhere to the provisions determined by the GDOC.) CQ1 targets individuals aged 18–64 years and CQ2 targets those aged ≥65 years.
(ii) Interventions: interventional studies that include exercise and physical activity. These interventions included both supervised and unsupervised exercise sessions. Exercise and physical activity interventions were defined according to the following definition of exercise stipulated in the MHLW’s Physical Activity Guideline for Health Promotion 2013: ‘Physical activity that is undertaken in a planned, deliberate and continuous manner with the aim of maintaining and improving physical fitness’. Examples of exercise include strength training and aerobics at a fitness club; sports, such as swimming, tennis and soccer; and activities, such as walking, jogging and hobbies, performed in one’s spare time. Lifestyle-related activities, such as daily work or household chores, and commuting to work or school are not included in this definition of exercise.
(iii) Comparator: no intervention (i.e. not engaging in exercise such as those listed above).
(iv) Outcomes: a total of 12 outcomes were proposed, so during a GDOC meeting, a vote was taken to determine their significance. The stipulated outcomes for each CQ were then selected based on the mean score of the votes of all attending GDOC members. The significant outcomes selected for CQ1 were survival, endurance, muscle strength, health-related QoL, cancer-related fatigue, exercise-related adverse events (AEs) and depression. In contrast, the significant outcomes selected for CQ2 were survival, health-related QoL, fatigue, depression, cognitive function, activities of daily living (ADLs) and exercise-related AEs.
(v) Language: English or Japanese.

##### Characteristics of studies identified in the literature searches

The literature searches identified a total of 1212 articles for CQ1, of which 370 were selected for secondary screening. Subsequent evaluation of these articles resulted in the selection of 74 articles as qualitative and quantitative systematic review targets (Supplementary Fig. 1a). The exercise interventions addressed in these target studies consisted of aerobic exercise, strength training, aerobic exercise combined with strength training and others (e.g. radio calisthenics, qigong, tai chi, gardening, group exercise, physical movement during daily activities and yoga).

The literature searches also yielded 903 articles as primary screening targets for CQ2, of which 370 were then selected as secondary screening targets. Subsequent evaluation of these articles resulted in the selection of 63 articles as qualitative systematic review targets and 40 articles as quantitative systematic review targets (Supplementary Fig. 1b). The exercise interventions addressed in these target studies consisted of aerobic exercise, strength training, aerobic exercise combined with strength training and others (e.g. radio calisthenics, qigong, tai chi, gardening, group exercise, physical movement during daily activities and yoga).

##### Recommendation strength and level of evidence

To develop the CPG recommendations, we conducted a systematic review focusing exclusively on RCTs, excluding existing CPGs, systematic reviews, open-label comparative studies and observational studies. The quality of the target studies was evaluated using the Cochrane Risk of Bias Tool, the results of which are presented in Supplementary Fig. 2. All methods for evaluating the body of evidence and for expressing the strength of that evidence adhered to the ‘MINDS Manual for Developing Clinical Practice Guidelines 2017’. Recommendations were adopted after considering factors, such as patient values, patient burden and risk of bias, in the studies subject to systematic review, with a focus on the level of evidence and the benefit–harm balance of significant outcomes of the CQ. The strength of each recommendation was indicated by an accompanying rating of the level of the evidence as either ‘strong’ (recommended) or ‘weak’ (suggested). For each clinical outcome evaluated in the systematic review, we provided an assessment of the level of evidence according to a structured grading system. The detailed evidence-grading criteria and outcome-specific certainty ratings are presented in Supplementary Table 2.

### Statistical analysis

Statistical analyses were performed using Review Manager version 5.41 (The Cochrane Collaboration, London, UK). Risk ratio (RR) and effect size (standardized mean difference; SMD) were calculated for each endpoint, and the magnitude of the effect for each study was expressed using a 95% confidence interval (CI). Fixed effect or random effect models were used to calculate these values depending on the extent of their heterogeneity. RR data based on the findings of individual studies and meta-analyses were visualized using forest plots. Heterogeneity was assessed using the *I*^2^ statistic. *Z*-test *P*-values under 0.05 were deemed to be statistically significant.

## Results

### Recommendations

**Figure 1:**
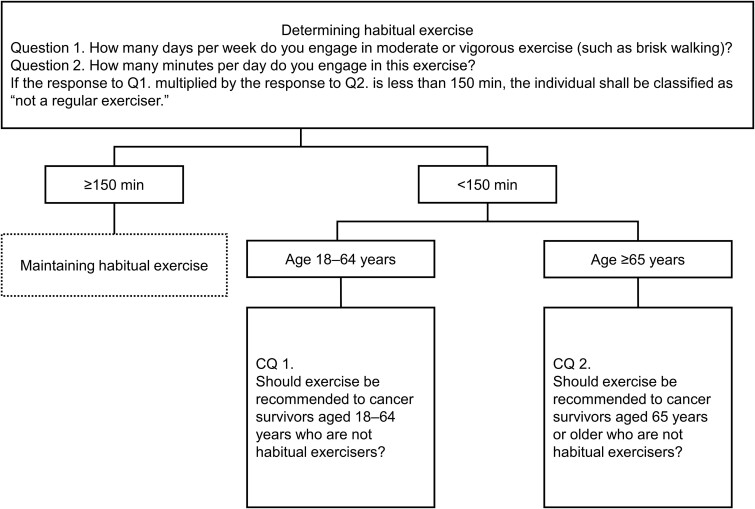
Exercise recommendation algorithm for cancer survivors.

Before presenting the recommendations, it is important to clarify the definition of habitual exercise used in these guidelines (Fig. 1). Recommendations apply to individuals who do not meet the following definition of habitual exercise.

Question 1: How many days per week do you engage in moderate or vigorous exercise (such as brisk walking)?

Question 2: How many minutes per day do you engage in this exercise?

If the product of the responses to Question 1 and Question 2 is <150 minutes per week, the individual is classified as ‘not a regular exerciser’.

### CQ 1

Should exercise be recommended to cancer survivors aged 18–64 years who are not habitual exercisers?

#### Recommendation

Exercise should be suggested to cancer survivors aged 18–64 years who are not habitual exercisers. Strength of recommendation: weak; level of evidence: C (weak).

#### Literature review and analysis

##### Survival—level of evidence: D (very weak)

The literature searches revealed only one RCT that had evaluated survival, so no meta-analysis was performed. The study (13) did not find any significant difference in the survival of the physical activity intervention group versus that of the control group (hazards ratio [HR] 0.86; 95% CI 0.35, 2.14).

##### Cardiorespiratory fitness—level of evidence: B (moderate)

Measures of endurance included maximum or peak oxygen uptake, estimated maximum oxygen uptake and 6-min walking distance. These measures used integrated scales to assess endurance. Meta-analysis of the 18 RCTs that assessed endurance (14–31) showed that exercise and physical activity interventions significantly improved endurance compared with the control group (SMD 0.65; 95% CI 0.34, 0.95) (Supplementary Fig. 3a).

##### Muscle strength—level of evidence: B (moderate)

Measures of muscle strength included maximum leg strength based on the assessment of maximum lifting weight, leg strength based on a simple method of assessing leg strength and grip strength. These measures used integrated scales to assess muscle strength. Meta-analysis of the nine RCTs that assessed muscle strength (17,18,23,24,27,30,32–34) revealed that exercise and physical activity interventions significantly improved muscle strength compared with the control group (SMD 0.79; 95% CI 0.22, 1.36) (Supplementary Fig. 3b).

##### Health-related QoL—level of evidence: B (moderate)

Measures of health-related QoL included the Functional Assessment of Cancer Therapy–General (FACT-G; including FACT-B and FACT-C), the European Organization for Research and Treatment of Cancer Quality of Life Questionnaire C30 (EORTC QLQ-C30), the Short Form-36 (SF-36) Physical Component Score (PCS) and the SF-36 Mental Component Score (MCS). As above, these measures used integrated scales to assess health-related QoL. Meta-analysis of the 18 RCTs that assessed QoL (17,19,21,24,26,28,29,31,35–45) showed that exercise and physical activity interventions significantly improved QoL compared with the control group (SMD 0.70; 95% CI 0.25, 1.14) (Supplementary Fig. 3c).

##### Fatigue—level of evidence: B (moderate)

Measures of fatigue included the Profile of Mood Scale (POMS), Piper Fatigue Scale (PFS), Functional Assessment of Chronic Illness Therapy-Fatigue Scale (FACIT-FS), Functional Assessment of Cancer Therapy-Fatigue (FACT-F), visual analog scale (VAS), Fatigue Severity Scale (FSS) and Schwartz Cancer Fatigue Scale (SCFS). These measures used integrated scales to assess fatigue. Meta-analysis of the 17 RCTs that assessed fatigue (15,17,24,26,28,30,32,36,37,39,42–49) showed that exercise and physical activity interventions significantly improved fatigue compared with the control group (SMD −0.50; 95% CI −0.74, −0.26) (Supplementary Fig. 3d).

##### Depression—level of evidence: C (weak)

Measures of depression included the Beck Depression Inventory version II (BDI-II), the Centers for Epidemiologic Studies-Depression Scale (CES-D) and the Hospital Anxiety and Depression Scale (HADS). These measures also used integrated scales to assess depression. Meta-analysis of the five RCTs that assessed depression (15,17,28,50,51) showed that exercise and physical activity interventions significantly improved depression compared with the control group (SMD −0.39; 95% CI −0.68, −0.10). The meta-analysis also revealed a low level of heterogeneity among studies, as indicated by the *I*^2^ value of 14% (Supplementary Fig. 3e).

##### Exercise-related AEs—level of evidence: B (moderate)

Only articles that reported incidences of AEs in a certain number of study participants were selected for the outcome of exercise-related AEs. Meta-analysis of the 21 RCTs that assessed exercise-related AEs (14,19,22,25–27,30,32,42,47,52–63) showed that exercise and physical activity interventions significantly increased the relative risk of exercise-related AEs compared with the control group (RR 2.83; 95% CI 1.79, 4.45) (Supplementary Fig. 3f). Incidences of exercise-related AEs included reports of muscle pain and other symptoms caused by exercise interventions, resulting in a high incidence proportion of these AEs in the intervention arm relative to their no-intervention counterparts. It is therefore conceivable that the studies describing such AEs only in the intervention group did not consider the issue of AEs in the no-intervention group. If AEs are designated as exercise-related AEs, the incidence proportion of AEs among the no-intervention group will naturally be 0 because they have not engaged in any exercise. Moreover, if reports of muscle pain are considered exercise-related AEs, there would be more instances of AEs following exercise than when no exercise is undertaken. However, the studies in question did not include serious AEs (SAEs) such as death and cardiovascular events caused by exercise.

#### Drafting the CQ1 recommendation

In drafting the CQ1 recommendation, improvements in endurance, health-related QoL, and fatigue due to exercise were emphasized. An outcome significance score of 8 (evidence level B) was assigned to QoL. In contrast, a score of 7 (evidence level D) was assigned to overall survival, and a score of 7 (evidence level B) was assigned to both endurance and fatigue. Although the evidence level for overall survival is D, an overall evidence level of C (‘weak’) was assigned, considering the significance of QoL. In addition to evidence level, discussions at the panel meetings to select the CQ1 recommendation focused primarily on assessing benefit–harm balance, patient values, patient burden and the risk of bias in studies subject to systematic review. For the assessment of benefit–harm balance, improvements in endurance, health-related QoL, and fatigue were emphasized as ‘beneficial’ outcomes, whereas AEs such as cardiovascular events were identified as ‘harmful’ outcomes. Specifically, these events were transient AEs that typically occurred as a result of exercise and that were only very rarely classified as SAEs. There were no AEs consisting of death or cardiovascular events due to exercise. Moreover, the desirable effects of exercise in terms of improved endurance, QoL and fatigue would likely outweigh the observed undesirable effects of exercise-induced AEs. The value assigned to outcomes deemed significant as well as the patient’s preferences and values concerning exercise are also likely to vary considerably. In terms of patient burden, although exercise does not impose a financial burden, the safe and effective practice of exercise might incur a financial burden, albeit not a major one. The final recommendation, ‘Exercise should be suggested to cancer survivors aged 18–64 years who are not habitual exercisers’, was adopted based on a 90% consensus among GDOC members.

### CQ 2

Should exercise be recommended to cancer survivors aged ≥65 years who are not habitual exercisers?

#### Recommendation

Exercise should be suggested to cancer survivors aged ≥65 years who are not habitual exercisers. Strength of recommendation: weak; level of evidence: B (moderate).

#### Literature review and analysis

##### Survival—level of evidence: D (very weak)

The literature searches revealed only one RCT that had evaluated survival, so no meta-analysis was performed. The study (13) did not find any significant difference in the survival of the physical activity intervention group versus that of the control group (HR 0.86; 95% CI 0.35, 2.14).

##### Health-related QoL—level of evidence: B (moderate)

Measures of health-related QoL included the FACT-G (including FACT-B and FACT-C), EORTC QLQ-C30, SF-36 (PCS) and SF-36 (MCS). These measures used integrated scales to assess health-related QoL. Meta-analysis of the 17 RCTs that assessed QoL (17,19,21,24,26,28,29,31,35–37,39–41,43–45) showed that exercise and physical activity interventions significantly improved QoL compared with the control group (SMD 0.70; 95% CI 0.23, 1.17) (Supplementary Fig. 4a).

##### Fatigue—level of evidence: B (moderate)

Measures of fatigue included POMS, PFS, FACIT-FS, FACT-F, VAS, FSS, SCFS and Fatigue Scale. These measures used integrated scales to assess fatigue. Meta-analysis of the 12 RCTs that assessed fatigue (17,24,26,28,30,36,37,39,43–45,49) showed that exercise and physical activity interventions significantly improved fatigue compared with the control group (SMD −0.30; 95% CI −0.53, −0.08) (Supplementary Fig. 4b).

##### Depression—level of evidence: C (weak)

Measures of depression included the BDI-II, CES-D and HADS. These measures also used integrated scales to assess depression. Meta-analysis of the four RCTs that assessed depression (17,28,50,51) showed that exercise and physical activity interventions significantly improved depression compared with the control group (SMD −0.39; 95% CI −0.74, −0.03) (Supplementary Fig. 4c).

##### Cognitive function—level of evidence: D (very weak)

The Functional Assessment of Cancer Therapy-Cognitive Function (FACT-Cog) perceived cognitive impairment subscale was selected for the assessment of cognitive function. The literature searches revealed only one RCT that had evaluated cognitive function (64), so no meta-analysis was performed. For the sake of reference, it is worth noting that the study reported a significant improvement in cognitive function in the physical activity intervention group compared with the control group (*P* = 0.01).

##### ADLs—level of evidence: unevaluable

The Disability Rating Index (DRI) sum score was selected for the assessment of ADLs. The literature searches revealed only one RCT that had evaluated ADLs (65), so no meta-analysis was performed. For the sake of reference, it is worth noting that the study reported a significant improvement in ADLs in the physical activity intervention group compared with the control group (*P* = 0.041).

##### Exercise-related AEs—level of evidence: B (moderate)

Only articles that reported incidences of AEs in a certain number of study participants were selected for the outcome of exercise-related AEs. Meta-analysis of the 19 RCTs that assessed exercise-related AEs (14,19,22,25–27,30,42,52–63) showed that exercise and physical activity interventions significantly increased the relative risk of exercise-related AEs compared with the control group (RR 2.77; 95% CI 1.72, 4.47) (Supplementary Fig. 4d). Incidences of exercise-related AEs included reports of muscle pain and other symptoms caused by exercise interventions, resulting in a high incidence proportion of these AEs in the intervention arm relative to their no-intervention counterparts. It is therefore conceivable that the studies describing such AEs only in the intervention group did not consider the issue of AEs in the no-intervention group. If AEs are designated as exercise-related AEs, the incidence proportion of AEs among the no-intervention group will naturally be 0 because they have not engaged in any exercise. Moreover, if reports of muscle pain are considered exercise-related AEs, there would be more instances of AEs following exercise than when no exercise is undertaken. However, the studies in question did not include SAEs such as death and cardiovascular events caused by exercise.

#### Drafting the CQ2 recommendation

In drafting the CQ2 recommendation, health-related QoL was assigned an outcome significance score of 8 (evidence level B), and fatigue was assigned a score of 7 (evidence level B). Based on the level of evidence for significant outcomes, the level of evidence was assessed as B (moderate). In addition to the level of evidence, discussions at the panel meetings to select the CQ2 recommendation focused primarily on assessing benefit–harm balance, patient values, patient burden and the risk of bias in studies subject to systematic review. For the assessment of benefit–harm balance, improvements in health-related QoL and fatigue were emphasized as ‘beneficial’ outcomes, whereas AEs such as cardiovascular events were identified as ‘harmful’ outcomes. Specifically, these events were AEs that typically occurred as a result of exercise and that were only rarely classified as SAEs. There were no AEs consisting of death or cardiovascular events due to exercise. Moreover, the desirable effects of exercise in terms of improved health-related QoL and fatigue would likely outweigh the observed undesirable effects of exercise-induced AEs. The value assigned to outcomes deemed to be significant as well as the patient’s preferences and values concerning exercise are also likely to vary considerably. In terms of patient burden, although exercise does not impose a financial burden, the safe and effective practice of exercise might incur a financial burden, albeit not a major one. The final recommendation, ‘Exercise should be suggested to cancer survivors aged ≥65 years who are not habitual exercisers’, was adopted based on a 90% consensus among GDOC members.

### External evaluation and public comment

The external evaluation was received in September 2023. The process of external evaluation was as follows. First, a draft of the CPG was circulated among the members of the External Evaluation Committee (EEC). Next, the Head of the CPG-DG provided a briefing of the draft document and responded to questions from the EEC members. After the briefing, the EEC members submitted comments. The CPG-DG revised the draft CPG based on these comments and reached a consensus on the revised content via email-based discussion in December 2023. That same month, the revised draft was circulated among EEC members, and the Head of the CPG-DG provided a briefing of the revised comments and responded to questions. The EEC members then submitted additional comments, but none of these warranted further changes to the revised draft. From February to March 2024, public comments were collected. The call for public comments was made through postings on the website of the National Cancer Center East Hospital and via email distribution to members of the Japanese Association of Supportive Care in Cancer. The CPG-DG finalized the draft based on a consensus reached through email-based discussions in April 2024.

## Discussion

It is important to note that according to the results of a systematic review of exercise intervention studies among the target population of cancer survivors (i.e. individuals aged ≥18 years who have been diagnosed with cancer and who are currently disease-free or who have stable disease), there have been few reports of AEs. However, the relative paucity of studies targeting individuals aged ≥65 years could also be considered a limitation of these Guidelines. The systematic review and meta-analyses performed during the development of the CPG did not make a clear distinction as to whether the target patients were aged ≤64 years or ≥65 years or older. The Guidelines took these limitations into account when assessing the benefit–harm balance and providing recommendations.

Functional assessments of older adult individuals have been identified as important tools in the US and are currently being implemented in Japan. Frailty health checkups are also being rolled out for older adults, but the task of performing these checkups has been entrusted to Japan’s local governments. There are presently no systems in place that are specifically geared towards older adults, so it is crucial to develop an environment in which such individuals can readily engage in exercise and physical activity.

In reviewing the participant data from the studies used to develop these guidelines, we found that the majority were breast cancer survivors (70.9%, *n* = 4682) and prostate cancer survivors (12.5%, *n* = 825). However, other cancer types were also represented, including colorectal (5.6%, *n* = 367), ovarian (2.4%, *n* = 159) and lung (1.5%, n = 99) cancer. A detailed distribution of the participants by cancer type is shown in Supplementary Table 3. This comprehensive dataset supports the relevance of the guidelines to a wide range of cancers beyond breast and prostate cancer. The principles of the guidelines, including personalized exercise prescriptions and consideration of individual health conditions, are designed to be broadly applicable and adaptable to the needs of survivors of different types of cancer. Therefore, while we acknowledge the predominance of breast and prostate cancer participants, we consider that these guidelines may offer a useful framework with potential applicability to a diverse population of cancer survivors.

Moreover, the scope of these guidelines does not extend to ‘individuals undergoing cancer pharmacotherapy (excluding maintenance chemotherapy for blood cancers and hormone therapy to prevent recurrence of breast, prostate and other cancers) or undergoing treatment for recurrent, metastatic or terminal cancers’. However, it is essential to organize the available evidence on exercise and physical activity intervention studies among populations not targeted by these guidelines, and we intend to address this need in future research.

In these guidelines, we developed recommendations that will enable healthcare professionals in clinical settings to provide exercise advice to cancer survivors. As such, the CPG does not contain suggestions on which specific types of exercise are preferable. The next version of the CPG will ideally contain specific recommendations on the types, intensity, duration and frequency of exercise. We will also investigate the feasibility of classifying studies according to cancer type and age (e.g. ≤64 years and ≥65 years), refining the scope of studies to those conducted in Asian populations, and performing subgroup analyses based on factors, such as monitored or unmonitored interventions and habitual or non-habitual exercisers.

## Conclusion

The consensus recommendation on exercise adopted in this CPG represents an important development in cancer survivorship care in Japan and is expected to contribute to improved clinical outcomes and advances in research. The CPG constitutes an important resource for both cancer survivors and clinicians. It emphasizes exercise as a key component of survivorship care and recommends appropriate physical activity to improve the health and wellbeing of cancer survivors.

## Supplementary Material

Supplementary_material_hyae126

## Data Availability

The data underlying this article are available in the article and in its online supplementary material.
