# Supplementary material for: Japan’s cancer survivorship guidelines for exercise and physical activity
Source: Jpn J Clin Oncol. 2024 Sep 19;55(1):12–20. doi: 10.1093/jjco/hyae126 (PMC11708214; doi:10.1093/jjco/hyae126)
Supplement: Supplementary_material_hyae126 [file supplementary_material_hyae126.zip › S-table1_hyae_126.docx]

**Supplementary Table 1.**

Search terms used into query PubMed

| Clinical Question | Search terms |
| --- | --- |
| CQ1 |  |
| #1 | Neoplasms[Mesh] |
| #2 | Cancer[TIAB] |
| #3 | Tumor[TIAB] |
| #4 | #1 OR #2 OR #3 |
| #5 | Exercise[Mesh] |
| #6 | Exercise therapy[Mesh] |
| #7 | #5 OR #6 |
| #8 | Survivor[Mesh] |
| #9 | surviv*[TIAB] |
| #10 | #8 OR #9 |
| #11 | #4 AND #7 AND #10 |
| #12 | randomized controlled trial[pt] OR controlled clinical trial[pt] OR randomized[tiab] OR placebo[tiab] OR drug therapy[sh] OR randomly[tiab] OR trial[tiab] OR groups[tiab] NOT (animals [mh] NOT humans [mh]) |
| #13 | 1966:2019/0/9/30[EDAT] |
| #14 | English[LA] OR Japanese[LA] |
| #15 | #11 AND #12 AND #13 AND #14 |
| #16 | #15 AND Filters: Adult: 19-44 years; Middle Aged: 45-64 years |
|  |  |
| CQ2 |  |
| #1 | Neoplasms[Mesh] |
| #2 | Cancer[TIAB] |
| #3 | Tumor[TIAB] |
| #4 | #1 OR #2 OR #3 |
| #5 | Exercise[Mesh] |
| #6 | Exercise therapy[Mesh] |
| #7 | #5 OR #6 |
| #8 | Survivor[Mesh] |
| #9 | surviv*[TIAB] |
| #10 | #8 OR #9 |
| #11 | #4 AND #7 AND #10 |
| #12 | randomized controlled trial[pt] OR controlled clinical trial[pt] OR randomized[tiab] OR placebo[tiab] OR drug therapy[sh] OR randomly[tiab] OR trial[tiab] OR groups[tiab] NOT (animals [mh] NOT humans [mh]) |
| #13 | 1966:2019/0/9/30[EDAT] |
| #14 | English[LA] OR Japanese[LA] |
| #15 | #11 AND #12 AND #13 AND #14 |
| #16 | #15 AND Filters: Aged: 65+ years |

CQ: clinical question.

Search terms used into query Cochran

| Clinical Question | Search terms |
| --- | --- |
| CQ1 |  |
| #1 | MeSH descriptor: [Neoplasms] explode all trees |
| #2 | (Cancer):ti,ab,kw |
| #3 | (neoplas*):ti,ab,kw |
| #4 | (Tumor*):ti,ab,kw |
| #5 | (Tumour*):ti,ab,kw |
| #6 | #1 or #2 or #3 or #4 or #5 |
| #7 | MeSH descriptor: [Exercise] explode all trees |
| #8 | MeSH descriptor: [Exercise Therapy] explode all trees |
| #9 | (physical activity):ti,ab,kw |
| #10 | (Exercise):ti,ab,kw |
| #11 | #7 or #8 or #9 or #10 |
| #12 | MeSH descriptor: [Survivors] explode all trees |
| #13 | (surviv*):ti,ab,kw |
| #14 | #12 or #13 |
| #15 | #6 and #11 and #14 |
| #16 | MeSH descriptor: [Adult] explode all trees |
| #17 | (adult*):ti,ab,kw |
| #18 | #16 or #17 |
| #19 | #15 and #18 with Publication Year to 2019, in Trials |
| #20 | #19 not pubmed:an |
|  |  |
| CQ2 |  |
| #1 | MeSH descriptor: [Neoplasms] explode all trees |
| #2 | (Cancer):ti,ab,kw |
| #3 | (neoplas*):ti,ab,kw |
| #4 | (Tumor*):ti,ab,kw |
| #5 | (Tumour*):ti,ab,kw |
| #6 | #1 or #2 or #3 or #4 or #5 |
| #7 | MeSH descriptor: [Exercise] explode all trees |
| #8 | MeSH descriptor: [Exercise Therapy] explode all trees |
| #9 | (physical activity):ti,ab,kw |
| #10 | (Exercise):ti,ab,kw |
| #11 | #7 or #8 or #9 or #10 |
| #12 | MeSH descriptor: [Survivors] explode all trees |
| #13 | (surviv*):ti,ab,kw |
| #14 | #12 or #13 |
| #15 | #6 and #11 and #14 |
| #16 | MeSH descriptor: [Aged] explode all trees |
| #17 | (aged):ti,ab,kw |
| #18 | #21 or #22 |
| #19 | #15 and #23 with Publication Year to 2019, in Trials |
| #20 | #24 not pubmed:an |

CQ: clinical question.

Search terms used into query 医中誌

| Clinical Question | Search terms | |  |
| --- | --- | --- | --- |
| CQ1 |  | |  |
| #1 | (癌サバイバー/TH or サバイバー/AL) | |  |
| #2 | がん患者/AL | |  |
| #3 | #1 OR #2 | |  |
| #4 | (身体運動/TH or 運動/AL) | |  |
| #5 | #3 AND #4 | |  |
| #6 | (#5) and (PT=会議録除く CK=成人(19～44),中年(45～64)) | |  |
|  |  |  |  |
| #1 | (腫瘍/TH or がん/AL) | |  |
| #2 | (身体運動/TH or 運動/AL) | |  |
| #3 | #1 and #2 | |  |
| #4 | (#3) and (RD=ランダム化比較試験,準ランダム化比較試験 CK=成人(19～44),中年(45～64)) | |  |
| #5 | (#4) and (PT=会議録除く) | |  |
|  |  | |  |
| CQ2 |  | |  |
| #1 | (癌サバイバー/TH or サバイバー/AL) | |  |
| #2 | がん患者/AL | |  |
| #3 | #1 OR #2 | |  |
| #4 | (身体運動/TH or 運動/AL) | |  |
| #5 | #3 AND #4 | |  |
| #6 | (#5) and (PT=会議録除く CK=高齢者(65～)) | |  |
|  |  | |  |
| #1 | (腫瘍/TH or がん/AL) | |  |
| #2 | (身体運動/TH or 運動/AL) | |  |
| #3 | #1 and #2 | |  |
| #4 | (#3) and (RD=ランダム化比較試験,準ランダム化比較試験 CK=高齢者(65～)) | |  |
| #5 | (#4) and (PT=会議録除く) | |  |

Search terms used into query SPORTDiscus

| Clinical Question | Search terms |
| --- | --- |
| CQ1 |  |
| #1 | DE "TUMORS" |
| #2 | TI cancer OR AB cancer |
| #3 | TI tumor OR AB tumor |
| #4 | (DE "TUMORS") OR ((TI cancer) OR (AB cancer)) OR ((TI tumor) OR (AB tumor)) |
| #5 | DE "EXERCISE" |
| #6 | DE "EXERCISE therapy" |
| #7 | (DE "EXERCISE" ) OR (DE "EXERCISE therapy") |
| #8 | DE "CANCER patients" |
| #9 | TI surviv* OR AB surviv* |
| #10 | (DE "CANCER patients") OR ((TI surviv*) OR (AB surviv*)) |
| #11 | ((DE "EXERCISE") OR (DE "EXERCISE therapy")) AND ((DE "CANCER patients") OR ((TI surviv*) OR (AB surviv*))) AND ((DE "TUMORS") OR ((TI cancer) OR (AB cancer)) OR ((TI tumor) OR (AB tumor))) |
| #12 | (SU randomized controlled trials OR (TI randomized OR AB randomized) OR (TI placebo OR AB placebo) OR SU drug therapy OR (TI randomly OR AB randomly) OR (TI trial* OR AB trial*) OR (TI group OR AB group)) NOT (SU animal NOT SU human) |
| #13 | 出版日付「1966年1月～2019年9月」 |
| #14 | LA English OR LA Japanese |
| #15 | (((DE "EXERCISE") OR (DE "EXERCISE therapy")) AND ((DE "CANCER patients") OR ((TI surviv*) OR (AB surviv*))) AND ((DE "TUMORS") OR ((TI cancer) OR (AB cancer)) OR ((TI tumor) OR (AB tumor)))) AND (SU randomized controlled trials OR (TI randomized OR AB randomized) OR (TI placebo OR AB placebo) OR SU drug therapy OR (TI randomly OR AB randomly) OR (TI trial* OR AB trial*) OR (TI group OR AB group)) AND (LA English OR LA Japanese)　+絞り込み出版日付「1966年1月～2019年9月」 |
|  |  |
| CQ2 |  |
| #1 | DE "TUMORS" |
| #2 | TI cancer OR AB cancer |
| #3 | TI tumor OR AB tumor |
| #4 | (DE "TUMORS") OR ((TI cancer) OR (AB cancer)) OR ((TI tumor) OR (AB tumor)) |
| #5 | DE "EXERCISE" |
| #6 | DE "EXERCISE therapy" |
| #7 | (DE "EXERCISE" ) OR (DE "EXERCISE therapy") |
| #8 | DE "CANCER patients" |
| #9 | TI surviv* OR AB surviv* |
| #10 | (DE "CANCER patients") OR ((TI surviv*) OR (AB surviv*)) |
| #11 | ((DE "EXERCISE") OR (DE "EXERCISE therapy")) AND ((DE "CANCER patients") OR ((TI surviv*) OR (AB surviv*))) AND ((DE "TUMORS") OR ((TI cancer) OR (AB cancer)) OR ((TI tumor) OR (AB tumor))) |
| #12 | (SU randomized controlled trials OR (TI randomized OR AB randomized) OR (TI placebo OR AB placebo) OR SU drug therapy OR (TI randomly OR AB randomly) OR (TI trial* OR AB trial*) OR (TI group OR AB group)) NOT (SU animal NOT SU human) |
| #13 | 出版日付「1966年1月～2019年9月」 |
| #14 | LA English OR LA Japanese |
| #15 | (((DE "EXERCISE") OR (DE "EXERCISE therapy")) AND ((DE "CANCER patients") OR ((TI surviv*) OR (AB surviv*))) AND ((DE "TUMORS") OR ((TI cancer) OR (AB cancer)) OR ((TI tumor) OR (AB tumor)))) AND (SU randomized controlled trials OR (TI randomized OR AB randomized) OR (TI placebo OR AB placebo) OR SU drug therapy OR (TI randomly OR AB randomly) OR (TI trial* OR AB trial*) OR (TI group OR AB group)) AND (LA English OR LA Japanese)　+絞り込み出版日付「1966年1月～2019年9月」 |
