# Supplementary material for: Japan’s cancer survivorship guidelines for exercise and physical activity
Source: Jpn J Clin Oncol. 2024 Sep 19;55(1):12–20. doi: 10.1093/jjco/hyae126 (PMC11708214; doi:10.1093/jjco/hyae126)
Supplement: Supplementary_material_hyae126 [file supplementary_material_hyae126.zip › S-table2_hyae_126.docx]

**Supplementary Table 2.** Level of Evidence by Outcome

| **Level of Evidence** | **Description** |
| --- | --- |
| A (strong) | There is strong confidence in the adequacy of the effect estimates to support the recommendation. |
| B (moderate) | There is moderate confidence in the adequacy of the effect estimates to support the recommendation. |
| C (weak) | There is limited confidence in the adequacy of the effect estimates to support the recommendation. |
| D (very weak) | There is little confidence in the adequacy of the effect estimates to support the recommendation. |

This table summarizes the level of evidence ratings for each clinical outcome, indicating the confidence level in the effect estimates used to support the recommendations.
