# Supplementary material for: Japan’s cancer survivorship guidelines for exercise and physical activity
Source: Jpn J Clin Oncol. 2024 Sep 19;55(1):12–20. doi: 10.1093/jjco/hyae126 (PMC11708214; doi:10.1093/jjco/hyae126)
Supplement: Supplementary_material_hyae126 [file supplementary_material_hyae126.zip › S-table3_hyae_126.docx]

**Supplementary Table 3.**

Participant Distribution by Cancer Type

| **Cancer Type** | **N** | **%** |
| --- | --- | --- |
| Acute Myeloid Leukemia | 38 | 0.6% |
| Breast | 4,682 | 70.9% |
| Cervix | 4 | 0.1% |
| Colon | 367 | 5.6% |
| Endometrial | 13 | 0.2% |
| Esophageal | 66 | 1.0% |
| Esophagogastric | 22 | 0.3% |
| Gastrointestinal | 2 | 0.0% |
| Genitourinary | 2 | 0.0% |
| Gynecologic | 41 | 0.6% |
| Hematologic | 4 | 0.1% |
| Lung | 99 | 1.5% |
| Lymphoma | 50 | 0.8% |
| Ovarian | 159 | 2.4% |
| Prostate | 825 | 12.5% |
| Rectum | 2 | 0.0% |
| Sarcoma | 2 | 0.0% |
| Testis | 136 | 2.1% |
| Thyroid | 4 | 0.1% |
| Mix | 64 | 1.0% |
| Other | 20 | 0.3% |
| **Total** | 6,602 | 100.0% |
