# Supplementary figures and images for: Japan’s cancer survivorship guidelines for exercise and physical activity
Source: Jpn J Clin Oncol. 2024 Sep 19;55(1):12–20. doi: 10.1093/jjco/hyae126 (PMC11708214; doi:10.1093/jjco/hyae126)

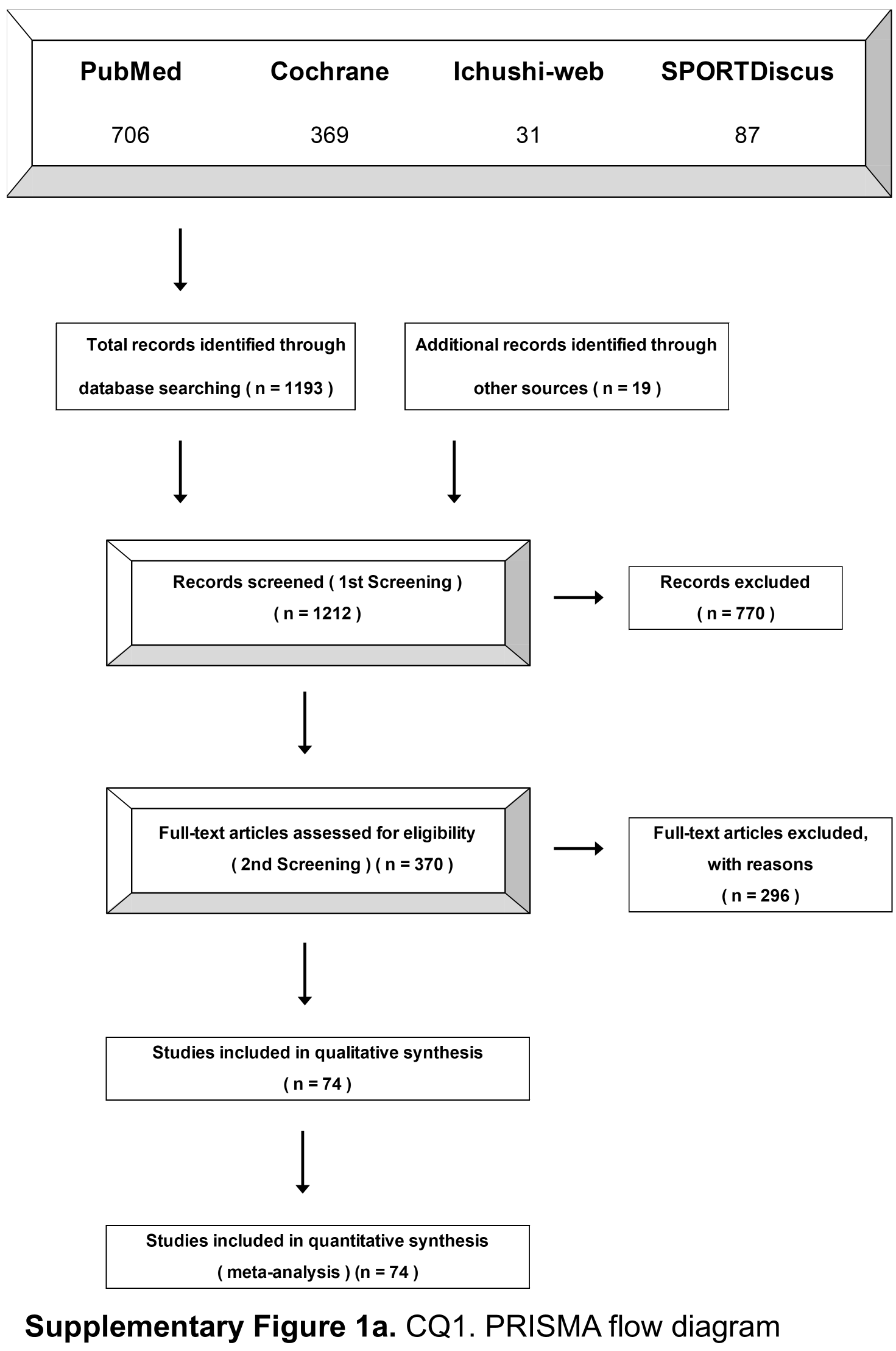

Supplement: Supplementary_material_hyae126 [file supplementary_material_hyae126.zip › S-Fig1a_hyae_126.TIF]

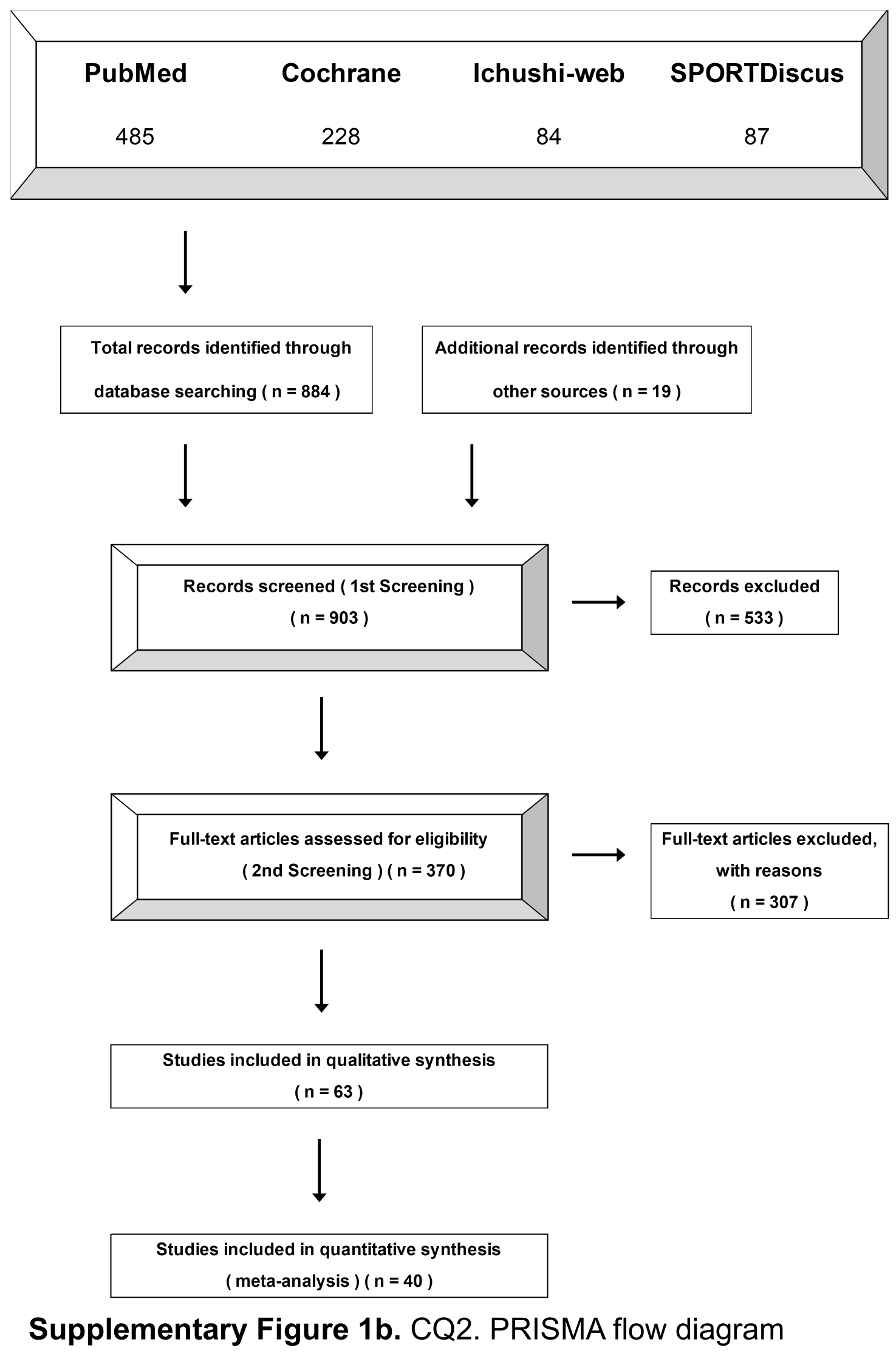

Supplement: Supplementary_material_hyae126 [file supplementary_material_hyae126.zip › S-Fig1b_hyae_126.TIF]

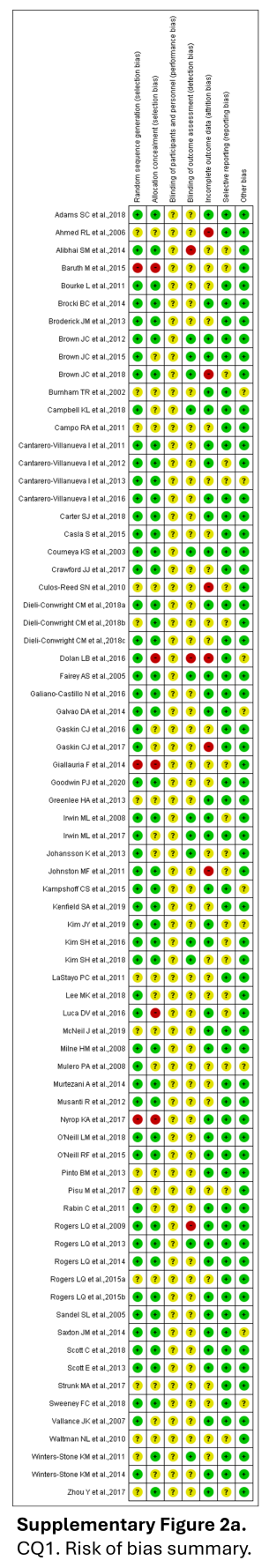

Supplement: Supplementary_material_hyae126 [file supplementary_material_hyae126.zip › S-Fig2a_hyae_126.TIF]

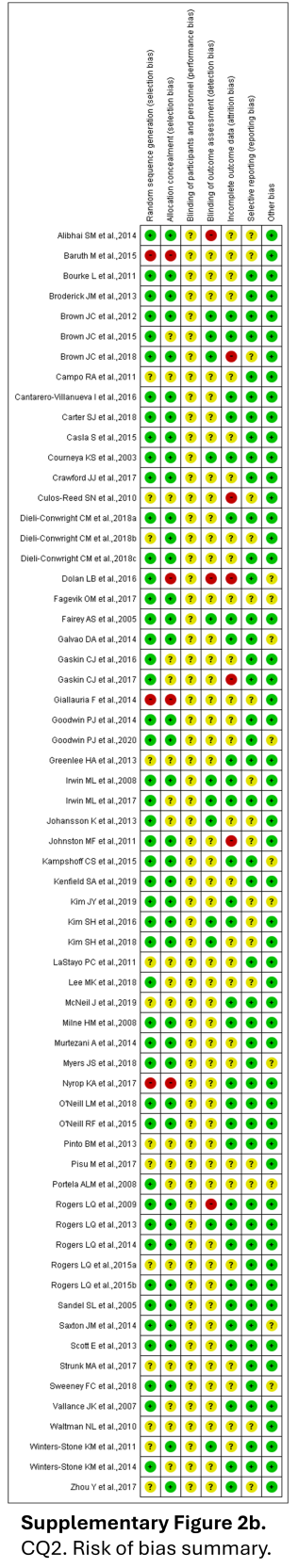

Supplement: Supplementary_material_hyae126 [file supplementary_material_hyae126.zip › S-Fig2b_hyae_126.TIF]

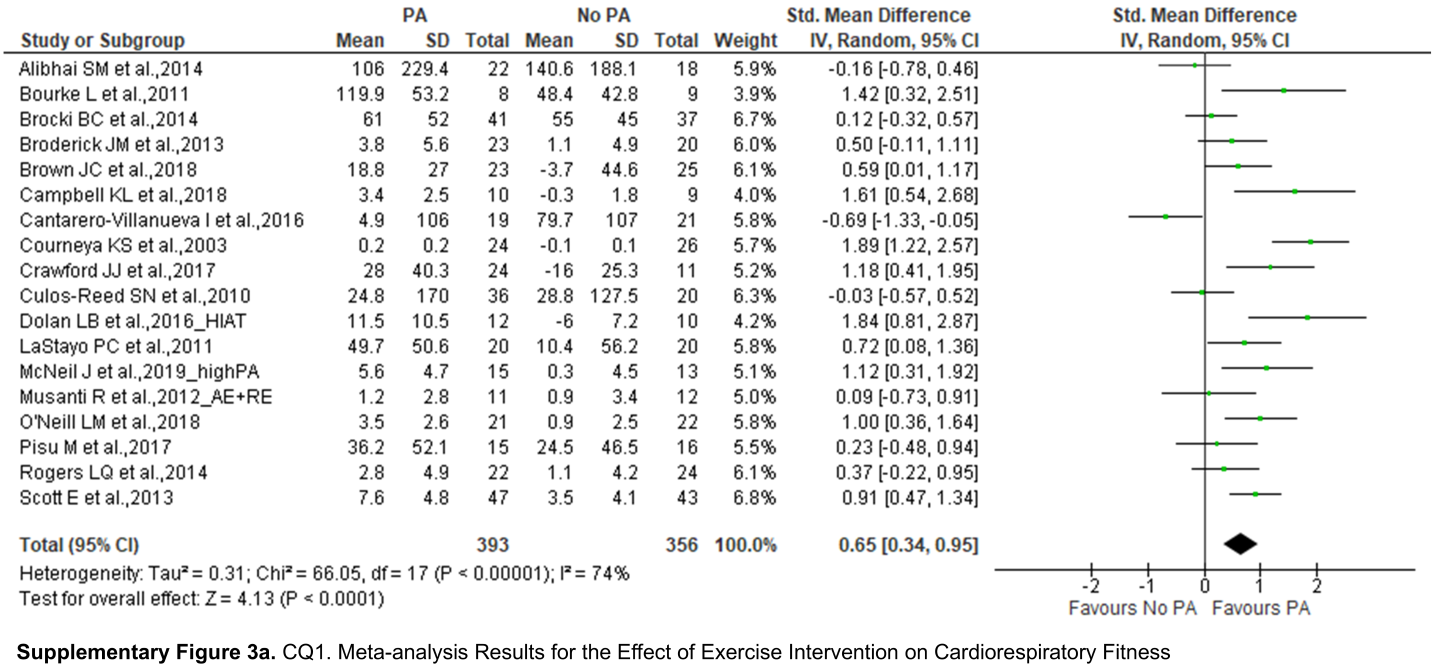

Supplement: Supplementary_material_hyae126 [file supplementary_material_hyae126.zip › S-Fig3a_hyae_126.TIF]

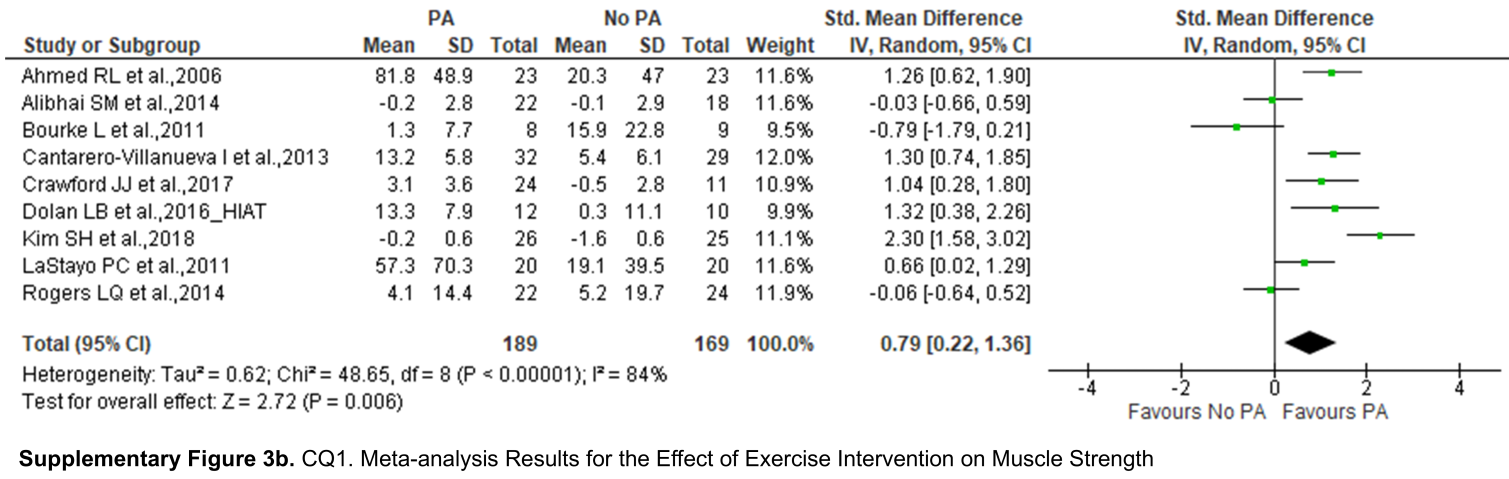

Supplement: Supplementary_material_hyae126 [file supplementary_material_hyae126.zip › S-Fig3b_hyae_126.TIF]

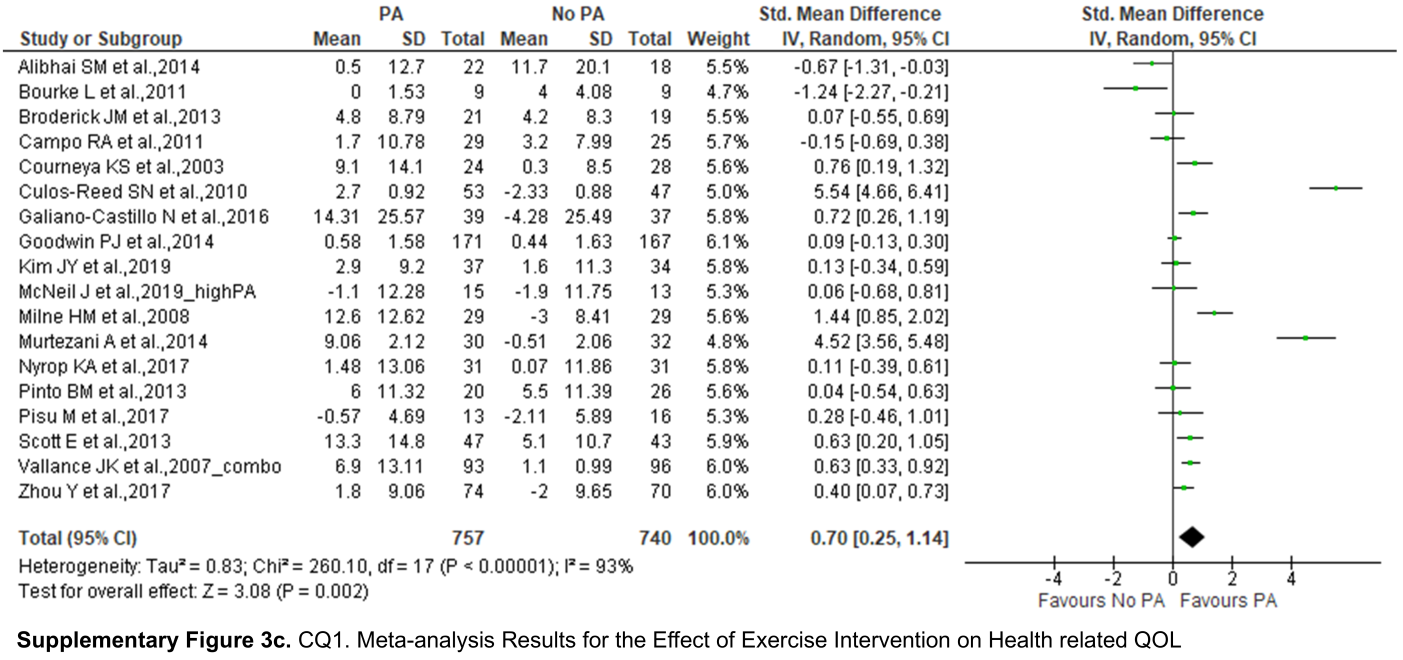

Supplement: Supplementary_material_hyae126 [file supplementary_material_hyae126.zip › S-Fig3c_hyae_126.TIF]

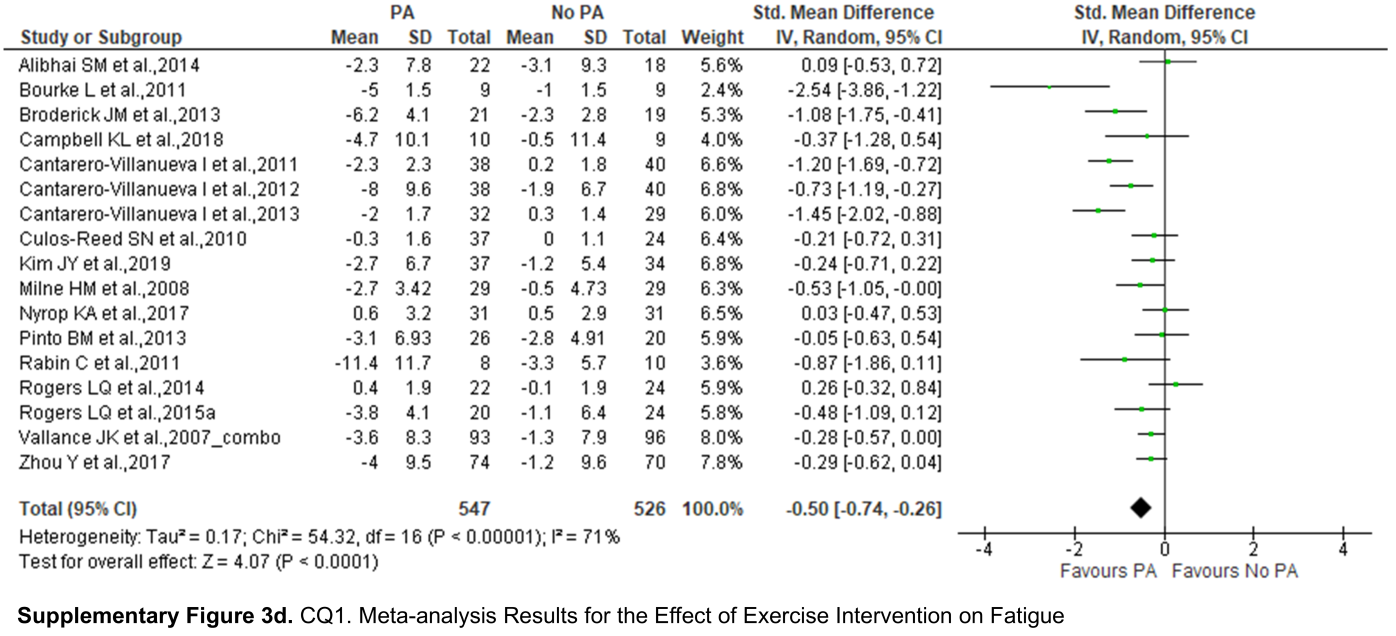

Supplement: Supplementary_material_hyae126 [file supplementary_material_hyae126.zip › S-Fig3d_hyae_126.TIF]

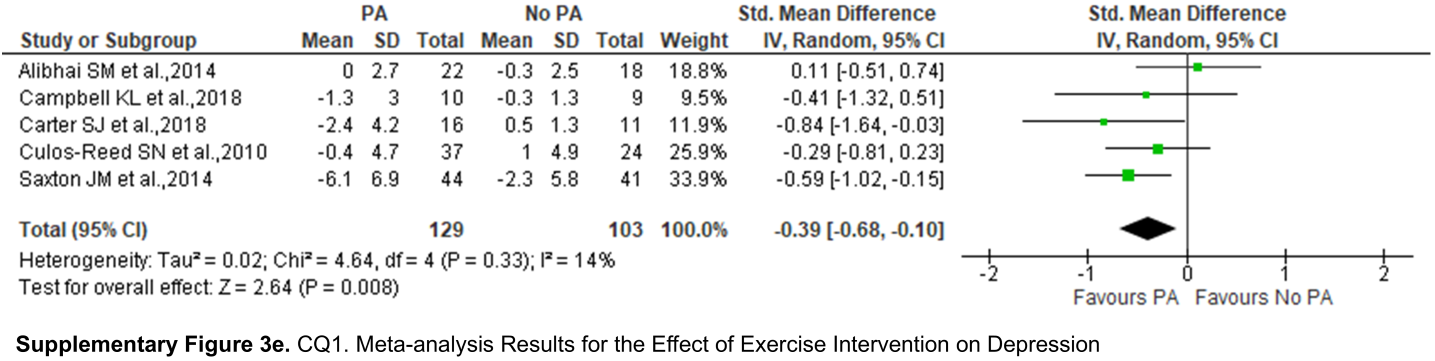

Supplement: Supplementary_material_hyae126 [file supplementary_material_hyae126.zip › S-Fig3e_hyae_126.TIF]

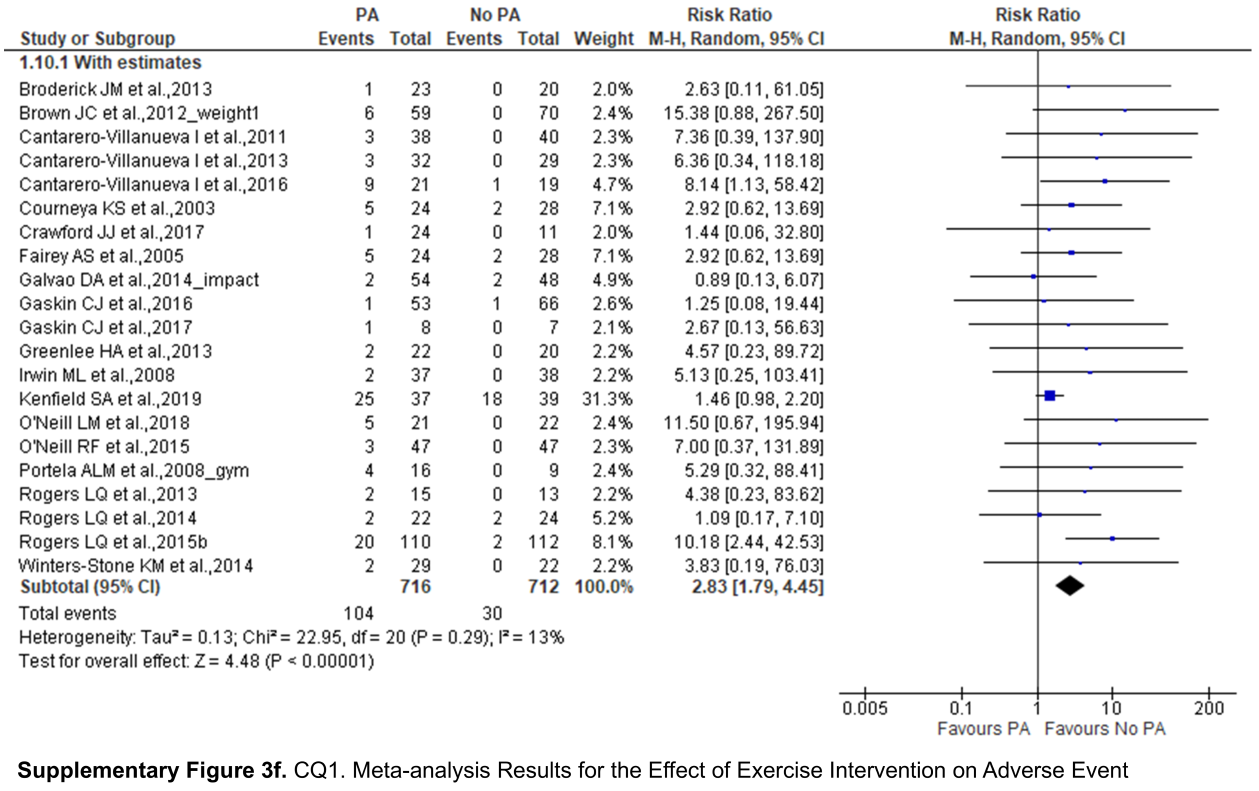

Supplement: Supplementary_material_hyae126 [file supplementary_material_hyae126.zip › S-Fig3f_hyae_126.TIF]

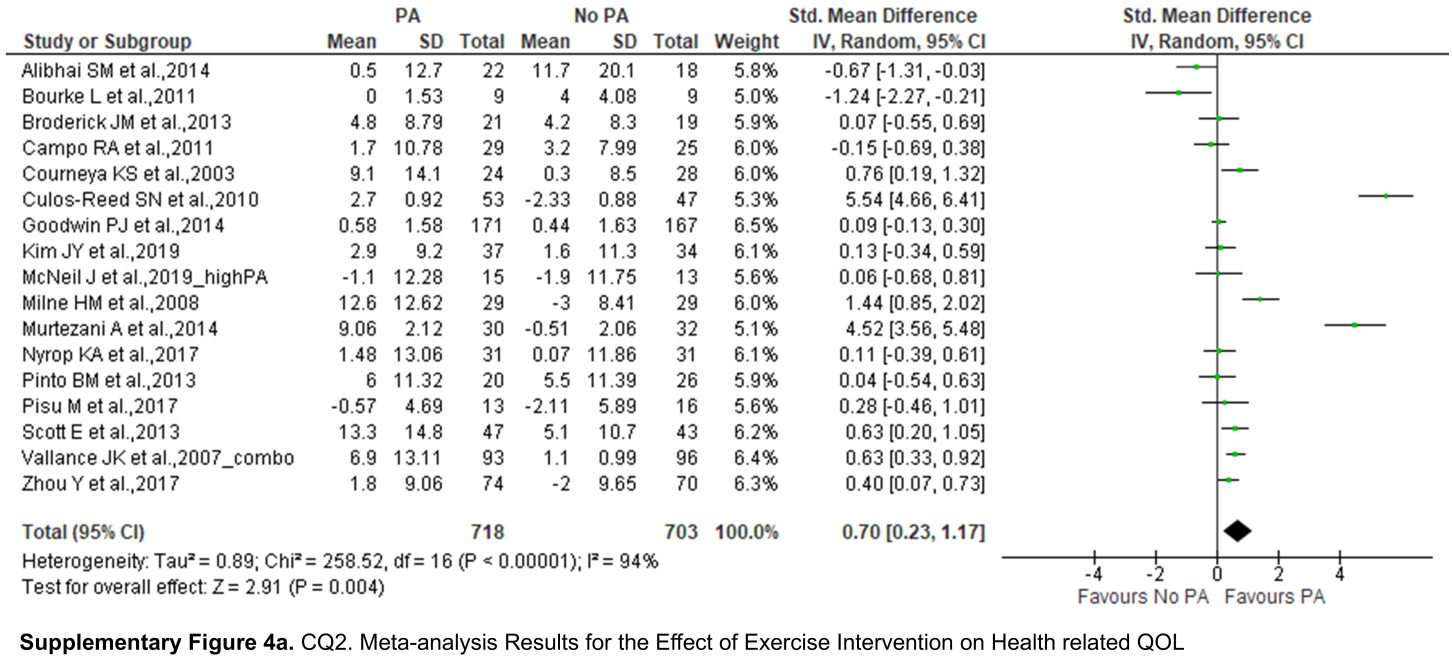

Supplement: Supplementary_material_hyae126 [file supplementary_material_hyae126.zip › S-Fig4a_hyae_126.TIF]

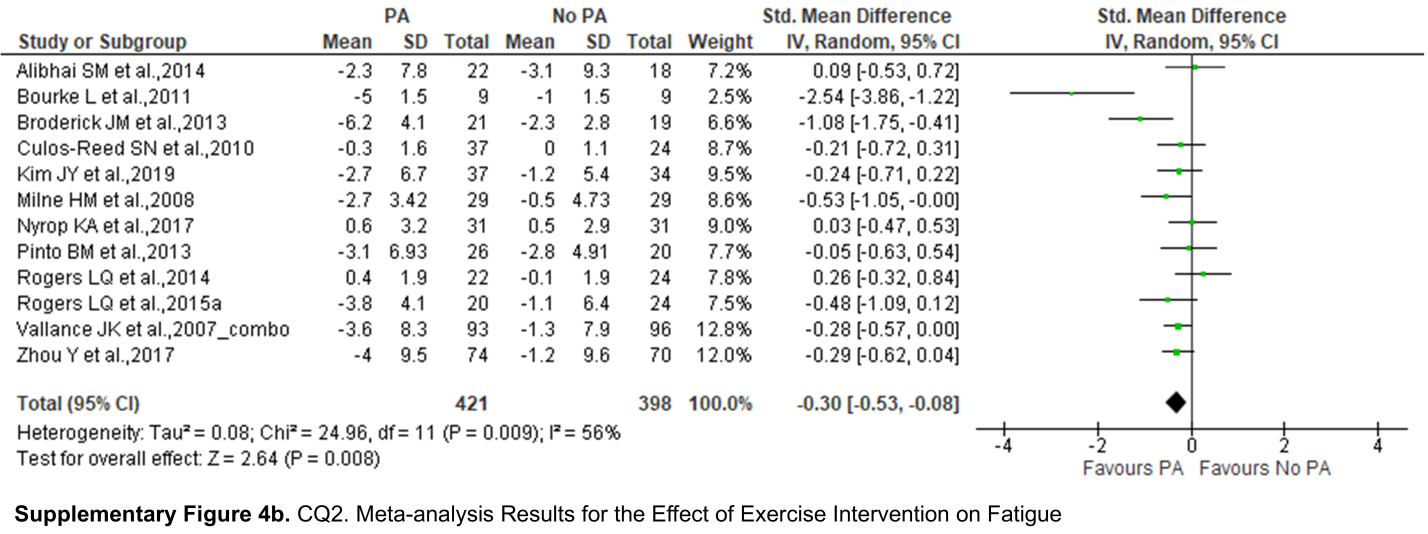

Supplement: Supplementary_material_hyae126 [file supplementary_material_hyae126.zip › S-Fig4b_hyae_126.TIF]

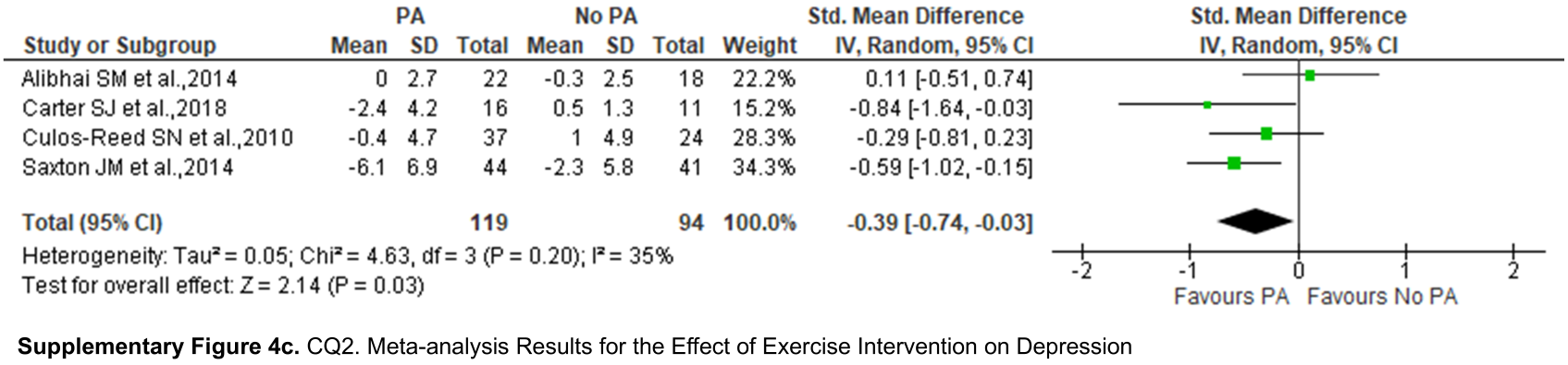

Supplement: Supplementary_material_hyae126 [file supplementary_material_hyae126.zip › S-Fig4c_hyae_126.TIF]

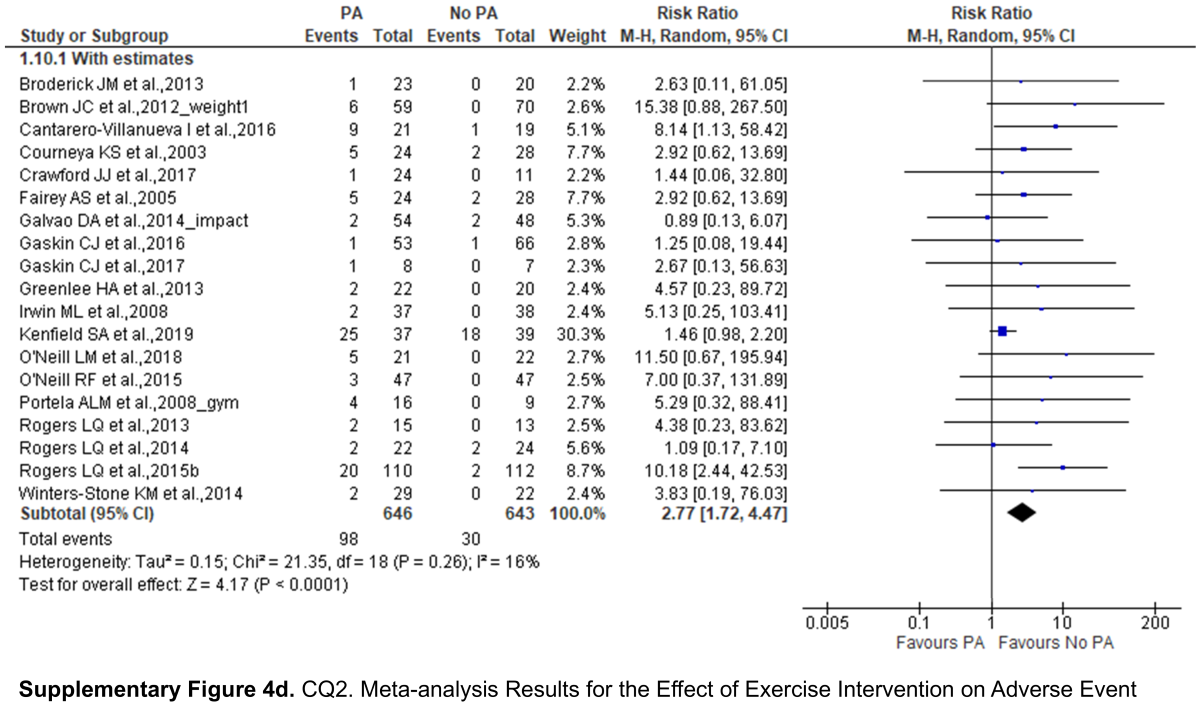

Supplement: Supplementary_material_hyae126 [file supplementary_material_hyae126.zip › S-Fig4d_hyae_126.TIF]
